# Comparative proteomics of the two *T*. *brucei* PABPs suggests that PABP2 controls bulk mRNA

**DOI:** 10.1371/journal.pntd.0006679

**Published:** 2018-07-24

**Authors:** Martin Zoltner, Nina Krienitz, Mark C. Field, Susanne Kramer

**Affiliations:** 1 School of Life Sciences, University of Dundee, Dundee, United Kingdom; 2 Department of Cell and Developmental Biology, Biocenter, University of Würzburg, Am Hubland, Würzburg, Germany; Liverpool School of Tropical Medicine, UNITED KINGDOM

## Abstract

Poly(A)-binding proteins (PABPs) regulate mRNA fate by controlling stability and translation through interactions with both the poly(A) tail and eIF4F complex. Many organisms have several paralogs of PABPs and eIF4F complex components and it is likely that different eIF4F/PABP complex combinations regulate distinct sets of mRNAs. Trypanosomes have five eIF4G paralogs, six of eIF4E and two PABPs, PABP1 and PABP2. Under starvation, polysomes dissociate and the majority of mRNAs, most translation initiation factors and PABP2 reversibly localise to starvation stress granules. To understand this more broadly we identified a protein interaction cohort for both *T*. *brucei* PABPs by cryo-mill/affinity purification-mass spectrometry. PABP1 very specifically interacts with the previously identified interactors eIF4E4 and eIF4G3 and few others. In contrast PABP2 is promiscuous, with a larger set of interactors including most translation initiation factors and most prominently eIF4G1, with its two partners TbG1-IP and TbG1-IP2. Only RBP23 was specific to PABP1, whilst 14 RNA-binding proteins were exclusively immunoprecipitated with PABP2. Significantly, PABP1 and associated proteins are largely excluded from starvation stress granules, but PABP2 and most interactors translocate to granules on starvation. We suggest that PABP1 regulates a small subpopulation of mainly small-sized mRNAs, as it interacts with a small and distinct set of proteins unable to enter the dominant pathway into starvation stress granules and localises preferentially to a subfraction of small polysomes. By contrast PABP2 likely regulates bulk mRNA translation, as it interacts with a wide range of proteins, enters stress granules and distributes over the full range of polysomes.

## Author summary

Poly(A)-binding proteins (PABPs) bind to the poly(A) tails of eukaryotic mRNAs and function in regulating mRNA fate. Many eukaryotes have several PABP paralogs and the current view is that each PABP binds a specific subset of mRNAs. *Trypanosoma brucei* has two PABPs, and to understand the differential functionality of these paralogs we identified interacting proteins for each. We found unique interactors for both PABPs, and significant differences between the two interaction cohorts. Our data indicate that the two PABP paralogs of trypanosomes have very distinct roles in mediating mRNA fate.

## Introduction

Gene expression is regulated by multiple transcriptional and post-transcriptional mechanisms. At the post-transcriptional level, regulation of protein synthesis by modulation of translation initiation is a major contributor. The first step in mRNA cap-dependent translation initiation is assembly of the eIF4F complex at the m^7^G cap of the mRNA 5’ end [1]. The eIF4F complex consists of a large (~180kDa) scaffold protein, eIF4G, bound to the cap-binding protein eIF4E and an RNA helicase, eIF4A. The latter is involved in secondary structure unwinding of the target mRNA, facilitating 40S subunit scanning, together with a further factor eIF4B. Significantly, eIF4G and eIF4B directly interact with the poly(A)-binding protein (PABP) associated with the poly(A) tail at the 3’ end of the target mRNA, to increase translation efficiency by mRNA circularisation and ribosome recycling.

Most higher eukaryotes have several paralogs of eIF4F complex subunits [2] and PABP [3]; increasing evidence suggests that these different paralogs can assemble into distinct eIF4F complexes, facilitating modulation of translation to distinct environmental and developmental conditions [4]. For example, in metazoa there is one eIF4F complex specialised to mediate cap-dependent translation under low oxygen conditions [5,6], and specific eIF4F complexes select distinct sets of mRNAs during development in *C*. *elegans* germ cells [7]. The specific functions of distinct eIF4F complexes are mediated by the properties of the individual subunits, for example *H*. *sapiens* eIF4E paralogs differ in their ability to localise to P-bodies and stress granules [8], ribonucleoprotein granules (RNA granules) with important functions in mRNA storage, regulation and quality control [9].

The presence of multiple PABP paralogs further increases the combinatorial complexity of this system. *Arabidopsis thaliana* has eight PABP paralogs [10] that differ in domain structure and expression patterns, with both overlapping and distinct functions [10–16]. *Xenopus laevis* has three paralogs that are all independently essential [17]. Many protozoa also possess several paralogs of each of the eIF4F complex subunits, but these are the product of lineage-specific expansions and hence unrelated to the paralogs found in higher eukaryotes. Very little is known about their specific functions [18,19].

Kinetoplastids, including the animal and human pathogens *Leishmania*, *Trypanosoma cruzi* and *T*. *brucei*, rely almost completely on post-transcriptional gene regulation [20]. mRNAs are transcribed poly-cistronically and processed by *trans*-splicing of a miniexon to the 5’ end, a process coupled to polyadenylation of the upstream transcript [21–26]. Furthermore, the mRNA cap structure is a highly unusual type four, with ribose 2’-O methylations at the first four transcribed nucleotides (AACU) and additional base methylations at the first (m_2_^6^A) and fourth (m^3^U) positions [27,28]. This cap requires a kinetoplastid-specific decapping enzyme for degradation [29]. Hence, translational control is a major contributor to gene regulation [30]. As a possible consequence of this kinetoplastids possess a large number of translation initiation factor paralogs [31]: six for eIF4E (eIF4E1-6), five for eIF4G (eIF4G1-5) and two for eIF4A (eIF4A1-2), of which only one, eIF4A1, is known to be involved in translation [32]. Trypanosomes have two PABP paralogs (PABP1, PABP2), while *Leishmania* has an additional paralog (PABP3).

Multiple studies have addressed the composition of kinetoplastid translation initiation complexes, and whilst data are equivocal in some cases, several distinct eIF4F complexes were described (recently reviewed in [31]. The best characterised complex comprises eIF4E4, eIF4G3, eIF4A1 and PABP1 in both *Leishmania* and trypanosomes [33–37]. Evidence of a direct physical interaction between eIF4E4 and eIF4G3 was obtained in *L*. *major* using yeast two hybrid [37], but direct binding between LmPABP1 and eIF4G3 was not observed [35,37]. Instead, LmPABP1 interacted directly with eIF4E4, mediated by the non-conserved N-terminal extension of eIF4E4 [35], an interaction critical for the function of eIF4E4 [38]. The current assumption that eIF4E4/eIF4G3/PABP1 is the major translation initiation complex is predicated on the following: i) all proteins are of high abundance, ii) PABP1 has greater specificity for poly(A) than PABP2 [36,39], iii) eIF4E4 binds the type 4 cap with the highest affinity of all eIF4E4 paralogs [40–42] and iv) silencing of eIF4E4, eIF4G3 and PABP1 in at least some *T*. *brucei* life cycle stages is lethal [33,34,36] and eIF4E4 cannot be deleted in *L*. *infantum* [38].

At least three additional translation initiation complexes are known. The first consists of eIF4E5 bound to either eIF4G1 or eIF4G2 [41]. Two further proteins specifically interact with the eIF4G1 version of this complex: TbG1-IP (Tb927.11.6720) and TbG1-IP2 (Tb927.11.350). TbG1-IP is an mRNA cap guanine-N7 methyltransferase, suggesting involvement in nuclear mRNA capping [43], but such a function is unlikely, as the protein is cytoplasmic and localises to starvation stress RNA granules [44], and nuclear cap methylation is known to be performed by TbCGM1 [45,46]. TbG1-IP2 is an RNA binding protein with unknown function. The second complex consists of eIF4G5, which specifically interacts with eIF4E6 and one further protein TbG5-IP (Tb927.11.14590) [42]. Interestingly, similarly to TbG1-IP1, this protein contains a nucleoside triphosphate hydrolase and a guanylyltransferase domain in common with enzymes involved in cap formation. The third complex consists of eIF4G4, eIF4E3 and eIF4A1 [33]. However, neither PABP was identified in any of these complexes.

Several studies have directly addressed function, substrate specificity and localisation of kinetoplastid poly(A)-binding proteins. PABP1 and PABP2 are highly abundant and in excess of the total number of mRNA molecules, at least in the procyclic life cycle stage of *T*. *brucei* [36]. RNAi in *T*. *brucei* revealed that both isoforms are essential [36] and both isoforms stimulate translation when tethered to the 3’ end of a reporter mRNA [47,48]. Both PABPs are cytoplasmic in untreated cells, but differentially localise under stress conditions: PABP2, but not PABP1, localises to the nucleus under certain conditions [36,49] and only PABP2 localises to starvation stress granules [49,50], while PABP1 and its interacting partners eIF4E4 and eIF4G3 do not [49]. Both PABPs localise to polysomes [49,51], but PABP1 is mainly located in small polysomes while PABP2 is more equally distributed across all polysomes [49]. There is some evidence that PABP2 may have a function unrelated to poly(A) binding. PABP2 binds poly(A) with lower specificity (in comparison to PABP1) in *Leishmania* [36,39] and binds to the CAUAGAAG element present in cell-cycle regulated mRNAs of *Crithidia fasiculata* [52] and to the U-rich RNA binding protein UBP1 [53], which mediates instability of the *T*. *cruzi* SMUG mucin mRNA [54].

To probe for distinct roles of PABPs we examined their protein interactomes in *T*. *brucei* procyclic forms. PABP1 co-precipitates eIF4E4 and eIF4G3 and RNA-binding protein RBP23, but few additional proteins. In contrast, PABP2 co-precipitated a large number of RNA binding proteins, including all proteins that co-precipitated with PABP1 except RBP23. Most eIF4F paralogs co-precipitated with PABP2, most significantly the eIF4G1/eIF4E5 complex and its two interacting partners TbG1-IP and TbG1-IP2. These data, together with analysis of the localisations of PABP1 and PABP2 complex components challenge the current paradigm that PABP1 is the major poly(A)-binding protein in trypanosomes and an alternative model is discussed.

## Results and discussion

### Purification of PABP complexes from *T*. *brucei*

**Fig 1:**
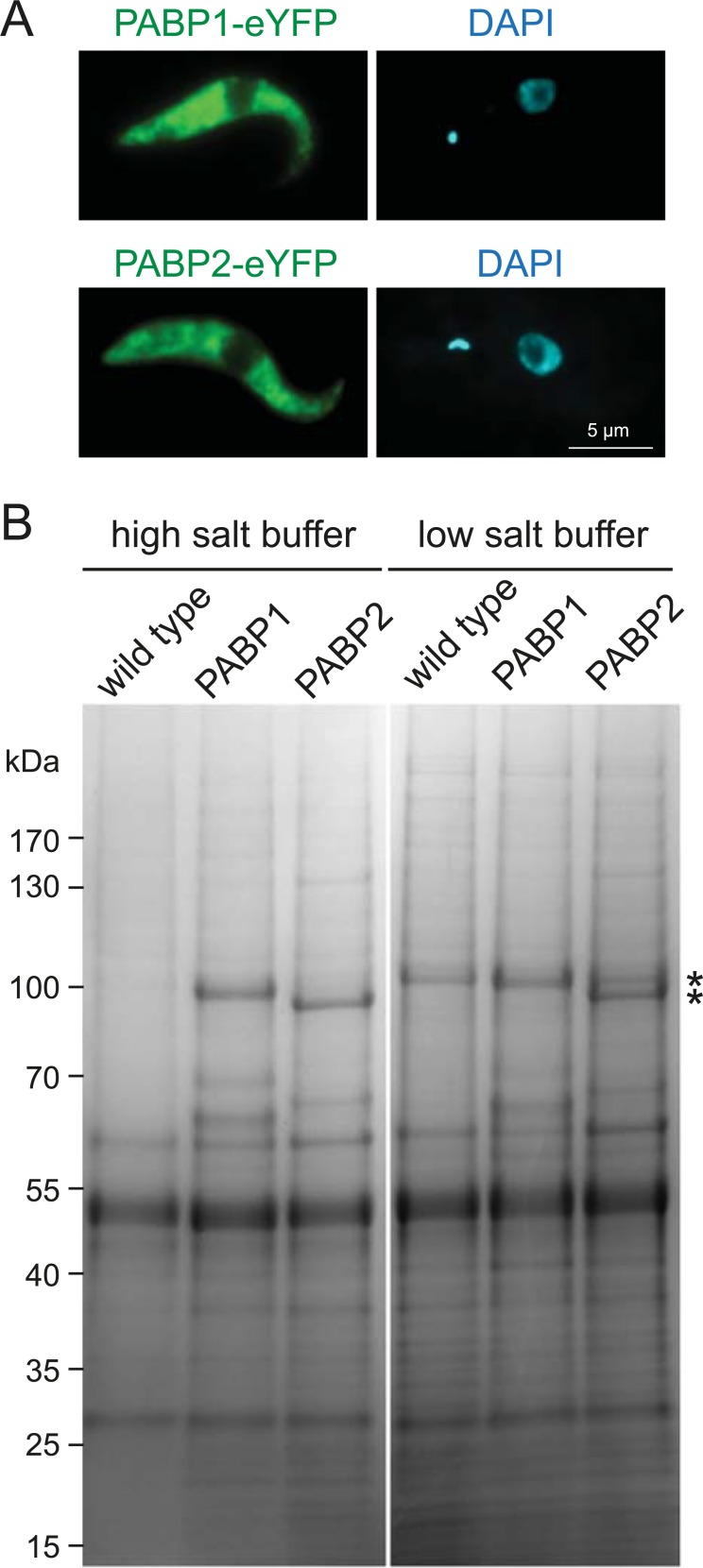
Isolation of PABP complexes. **(A)** Z-stack projection image of a representative trypanosome cell expressing PABP1-eYFP or PABP2-eYFP from endogenous loci. **(B)** Proteins were immunoprecipitated with polyclonal anti-GFP Llama antibodies coupled to Dynabeads. Representative Coomassie stained polyacrylamide gels loaded with immunoprecipitate from wild type cells (control), cells expressing PABP1-eYFP and cells expressing PABP2-eYFP are shown, for isolations performed in high salt and low salt buffers (see methods). The theoretical molecular weights of the bait proteins, PABP1-eYFP and PABP2-eYFP, are 95.6 and 94.7 kDa, respectively (asterisk). Note that with the low salt buffer, a nonspecific band migrates at almost identical position to PABP1-eYFP; the PABP1-eYFP band in lane five is slightly wider, indicating a doublet with the nonspecific protein (top) and PABP1-eYFP (bottom).

To isolate PABP complexes we used two previously published cell lines expressing C-terminal eYFP fusions of each PABP paralog from their endogenous locus; the second allele remained unaltered [49]. PABP1-eYFP is fully functional as deletion of the wild type allele has no phenotype, while RNAi that targets both alleles is lethal. In the cell line expressing PABP2-eYFP, the second allele could not be deleted, but cells have normal growth rates and localisation of the protein to various types of RNA granules was indistinguishable from that determined with specific antiserum against PABP2 [49]. This indicates that most functions of PABP2-eYFP are essentially identical to the wild type protein. Protein expression and localisation to the cytoplasm was demonstrated by fluorescence microscopy (Fig 1A). Wild type cells served as negative controls.

Cultures of each cell line were snap-frozen and subjected to cryomilling to generate a powder [55]. Aliquots of this powder were used to systematically optimise conditions for isolation of PABP complexes. Protein complexes were captured with polyclonal anti-GFP antibodies covalently coupled to magnetic Dynabeads and analysed by SDS-PAGE. In the optimised protocol, the cell powder was solubilised using CHAPS detergent with two different buffers: a low salt buffer and a high salt buffer, the latter contained 150 mM KCl but was otherwise identical to the low salt buffer. Coomassie-stained gels revealed clearly visible PABP bait proteins and several protein bands specific to one or both PABPs, but absent from the control pull down (Fig 1B).

For each cell line, protein complexes were isolated in two independent experiments for each buffer condition and the captured proteins analysed by liquid chromatography tandem mass spectrometry (LC-MS^2^) and subjected to label free quantification using MaxQuant [56]. 1901 distinct protein groups (peptides assigned to a specific coding sequence, but where these cannot be assigned to a single gene in the case of close paralogs) were identified (S1 Table); this list was reduced to 1224 after removing all protein groups with less than three unique peptides (S1B Table). For each protein group from each experiment we determined the enrichment ratio in relation to the wild type control cell line, based on quantification by unique peptides only. To avoid division by zero, a constant (0.001) was added to each LFQ value; such ‘infinite ratios’ are clearly distinguishable from genuine ratios by being significantly larger, smaller or exactly 1.0 (S1B Table).

For PABP1, we identified 25 proteins at least two-fold enriched in each of the two low salt replicates (S1C Table) and 66 proteins at least two-fold enriched in both high salt replicates (S1D Table). For PABP2, 77 and 170 proteins were enriched in both replicates under low salt and high salt conditions, respectively (S1E and S1F Table). Ribosomal proteins were exclusively co-precipitated under high salt conditions and not detected under low salt, consistent with intact ribosomes requiring physiological potassium concentrations and dissociating upon potassium depletion [57,58]. Interestingly, the number of co-precipitated ribosomal proteins differed between the PABP1 and PABP2 pull-downs: 43 proteins were co-purified with PABP2 (25% of all precipitated proteins), but only 7 ribosomal proteins with PABP1 (11% of all precipitated proteins). This could reflect differences in polysomal association between the two isoforms: PABP2 associates with heavier sucrose fractions than PABP1 on polysome fractionation gradients [49]. Alternatively, these differences could be explained by the RNA-binding ability of PABP2 being less specific to poly(A) tails in comparison to PABP1, as has been previously found for *Leishmania* orthologues [36]: unspecific binding of PABP2 to ribosomal RNA could cause co-precipitation of intact ribosomes under high salt conditions, resulting in the presence of ribosomal proteins in the proteomics data. Evidence for the second hypothesis is provided by the large number of nucleolus-localised proteins in the PABP2 pull-down with high salt buffer: 20 of the 127 non-ribosomal proteins purified with PABP2 are known to entirely or predominantly localise to the nucleolus, in comparison to only 4 of 59 non-ribosomal proteins purified with PABP1 [59]. PABP2 does not localise to the nucleolus, at least not to detectable levels, thus, these interactions are likely non-physiological.

All PABP interacting proteins were judged for their possible function in mRNA metabolism. A protein was classified as having a known role in mRNA metabolism (indicated as ‘yes’ in S1C–S1F Table), if it possesses an RNA-binding domain, or if there is direct experimental evidence for involvement in RNA metabolism (for example validated localisation to RNA granules). A protein was classified as having a predicted role in mRNA metabolism (indicated as ‘(yes)’ in S1C–S1F Table) if it was identified in one out of three large scale experiment that screened for posttranscriptional activators, repressors and RNA-binding proteins [47,48], without further experimental validation. The low salt precipitations contained mostly proteins with a known or predicted function in mRNA metabolism for both PABP1 (19/25 proteins) and PABP2 (62/77 proteins) and few obvious contaminants. High salt precipitations were still enriched in mRNA metabolism proteins (PABP1 28/66 and PABP2 54/170) but also contained a large fraction of likely or obvious contaminants, including mitochondrial, nucleolar and ribosomal proteins.

### A high confidence list of PABP-interacting proteins

**Fig 2:**
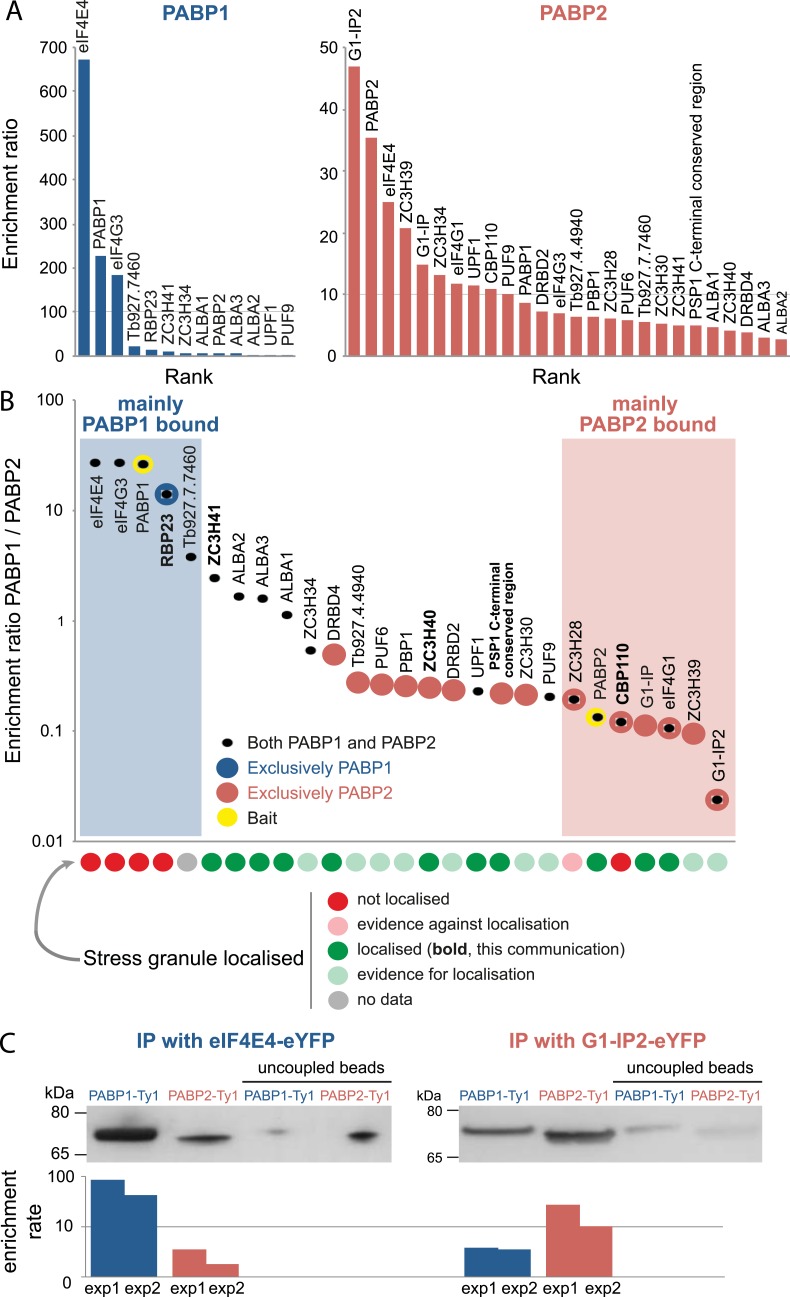
Proteins interacting with PABP1 and PABP2. A high confidence list of PABP interacting proteins was obtained by excluding proteins with an enrichment score less than two-fold in all four replicates; moreover, maximal one infinite ratio was tolerated. **(A)** Proteins co-precipitating with PABP1 (blue) or PABP2 (red) are shown sorted according to their average enrichment rates. Note the differences in scale on the y-axis between the two PABPs. **(B)** Differences between the interactomes of PABP paralogs; all 27 proteins that are enriched in either or both PABP pull-downs are ranked according to enrichment ratio PABP1/PABP2. Bait proteins are shown in yellow. Proteins unique to either PABP, defined by having an enrichment ratio <2 in the other PABP, are shown as large circles. Localisation to starvation stress granules is indicated for each protein (see S1G Table for references). Stress granule localisation information based on experimental data are shown in dark colours, other evidence (from TrypTag data [59]) is in lighter colours. **(C)** Reverse immunoprecipitations: eIF4E4 or G1-IP2 was expressed as an eYFP fusion protein together with either PABP1-4Ty1 or PABP2-4Ty1, all from their endogenous locus. eIF4E4 and G1-IP2 were precipitated from cryo-milled powders with recombinant anti-GFP Llama antibodies. Co-precipitated PABP proteins were detected with anti-Ty1 (BB2) monoclonal antibody. Beads without antibody were employed to calculate the enrichment factor. Data from two replicates (exp1 and exp2) are shown, and one representative blot.

To obtain a high confidence list, we filtered for proteins that were at least two-fold enriched in all four experiments. In a second step, all protein groups with more than one infinite ratio were removed, and three further proteins were manually removed because they were obvious contaminations; two mitochondrial RNA-binding proteins (Tb927.7.2570, Tb927.2.3800) and one glycosomal protein (Tb927.10.5620). Average enrichment ratios were calculated for each protein, excluding ‘infinite ratios’ (S1G Table, Fig 2A) together with a PAPB1/PABP2 enrichment ratio, to determine the specificity of each interaction (S1G Table, Fig 2B).

All 27 PABP-interacting proteins have a known or predicted function in mRNA metabolism. 12/27 proteins were more than 2-fold enriched in both pull-downs. For 6 of the 27 proteins the interaction with PABP(s) had been independently validated in at least one of the Kinetoplastids: ALBA1-3 co-precipitate both *T*. *brucei* PABPs [60]. Both *T*. *brucei* PABPs were found in a large scale yeast 2-hybrid screen to interact with PBP1 [61]. Several studies have identified PABP1 as part of the eIF4G3/eIF4E4 complex in *Leishmania* [34–36], with an unusual direct interaction between PABP1 and eIF4E4 [35]. For *Leishmania* eIF4E4, additional interaction with PABP2 was shown [35]. Moreover, while this manuscript was in revision, a PABP1 interactome for *Leishmania infantum* was published [62] and is in agreement with our data: seven proteins consistently co-precipitated with *Leishmania* PABP1, of which six correspond to the six most enriched proteins in the *T*. *brucei* PABP1 pulldown (eIF4E4, eIF4G3, PABP1, RBP23, Tb927.7.7460, ZC3H41) and only one protein (Tb927.10.13800) was not identified with our conditions. As a further control, we performed reverse pull-downs of the proteins mostly enriched in either the PABP1 pull-down (eIF4E4) or the PABP2 pull-down (G1-IP2) (Fig 2C). For this, eIF4E4 and G1-IP2 were expressed as eYFP fusion proteins from their endogenous loci, in cell lines also expressing PABP1 or PABP2 C-terminally fused to a tandem of four Ty1 epitopes. Precipitations of eIF4E-eYFP and G1-IP2-eYFP were performed as above, using low salt buffer conditions. Co-precipitated PABP-Ty1 proteins were detected by western blot probed with anti-Ty1. Both PABP proteins were enriched in these pull-downs in comparison to the negative control. However, in agreement with the mass spectrometry, PABP1 had a much higher enrichment ratio than PABP2 in the eIF4E4 pull-down (64-fold/3-fold on average for PABP1/PABP2, n = 2) while the opposite was found for the G1-IP2 pull-down (4-fold/19-fold on average for PABP1/PABP2, n = 2).

### Proteins specific to either PABP

Two proteins were particularly enriched in the PABP1 pull-down, with a more than 100-fold enrichment and more than 20-fold enrichment against PABP2. These are the two known PABP1 interactors eIF4E4 and eIF4G3, confirming the specificity of the pull-down. Only three further proteins had average enrichment ratios of >10 in comparison to the negative control, namely the RNA binding protein RBP23, a hypothetical protein Tb927.7.7460 and the CCCH type zinc finger protein ZC3H41; all experimentally uncharacterised. RBP23 was the only protein that was solely precipitated with PABP1: all other PABP1 interacting proteins also interact with PABP2, *albeit* in most cases with lower enrichment ratios.

**Fig 3:**
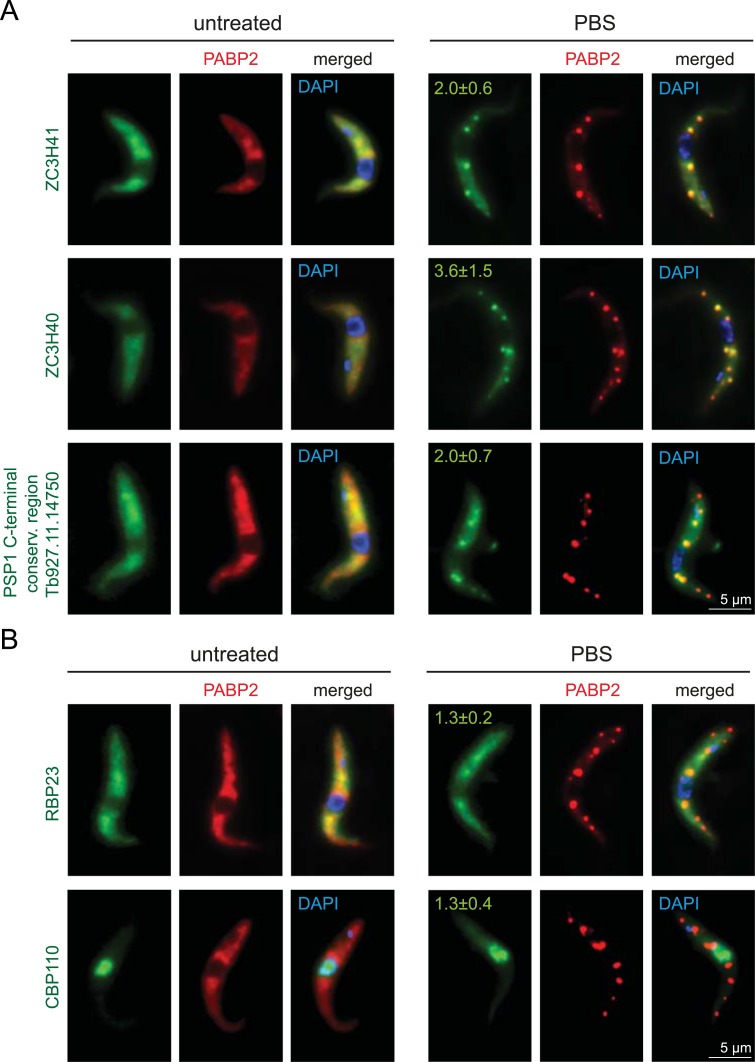
Localisation of PABP-interacting proteins to starvation stress granules. PABP interacting proteins were expressed as eYFP fusion proteins in a cell line expressing the starvation stress granule marker PABP2 as a C-terminal mChFP fusion. One representative Z-stack projection image is shown for untreated and starved parasites for proteins that localise to granules **(A)** and proteins that are (largely) absent from granules **(B)**. For all PBS treated cells, the average stress granule enrichment ratio with standard deviation is shown on top of each image: Granules were defined on the mChFP image (PABP2) by threshold settings using the maximal entropy method of ImageJ (https://imagej.nih.gov/ij/) and the background corrected fluorescence within the granule divided by the background corrected fluorescence next to the granule was calculated for the three largest granules of ten cells for all five eYFP fusion proteins. Broad-field images of all cell lines are available in S1 Fig.

In contrast, PABP2 co-precipitated a larger number of proteins than PABP1, but with much lower enrichment ratios, possibly reflecting greater promiscuity and interactions with a larger number of heterogenic target mRNAs and hence likely representing isolation of multiple PABP2 complexes. Of the seven proteins most specific to the PABP2 pull-down, three were members of the previously characterised eIF4G1/eIF4E4 complex [41], namely eIF4G1, the RNA-binding protein Tb927.11.350 (G1-IP2) and Tb927.11.6720, an mRNA cap guanine-N7 methyltransferase. One of the specific PABP2 targets with high enrichment ratio, CBP110, is localised to the nucleoplasm [63] (Fig 3B); this could be a true interacting protein given that PABP2 shuttles between the nucleus and the cytoplasm [36,49]. Among the PABP2 interacting proteins were 14 proteins that had enrichment rates of <2 in the PABP1 pull-down and thus appeared specific to PABP2 (Fig 2B).

### PABP complexes and RNA granules

As we observed major differences between the two PABPs in localisation to RNA granules, we analysed the localisation to RNA granules for all 27 proteins that interact with either or both PABPs (Fig 2B, S1G Table). We used published data that used either DHH1, PABP2 or poly(A) as stress granule markers [44,49,60,64] or co-expressed several proteins as eYFP fusions with a mChFP fusion of the stress granule marker protein PABP2 (Fig 3 and S1 Fig). In addition, we obtained information from the genome-tagging project TrypTag [59] (http://www.tryptag.org, with permission). For the TrypTag project, cells are washed in amino acid free buffer prior to imaging and starvation stress granules are therefore visible. The majority of proteins (20/27) localised to RNA granules, for one protein the localisation remains unknown as tagging failed, and only six proteins did not localise to RNA granules. At least four of the five proteins mostly enriched in the PABP1 pull-down were excluded from granules; these include the unique PABP1-interacting protein RBP23 (Fig 3A and S1D Fig). In contrast, for the majority of the PABP2-interactors there is evidence or proof for stress granule localisation. Only two proteins of the PABP2 interacting proteins are excluded from granules, one is the nuclear protein CBP110, which is not expected to localise to RNA granules and the other the zinc finger protein ZC3H28. Thus, the PABP1 complex appears largely excluded from granules, while most of the PABP2 interacting proteins localise to granules, similar to the majority of mRNAs [44].

### PABPs and translation initiation complexes

**Fig 4:**
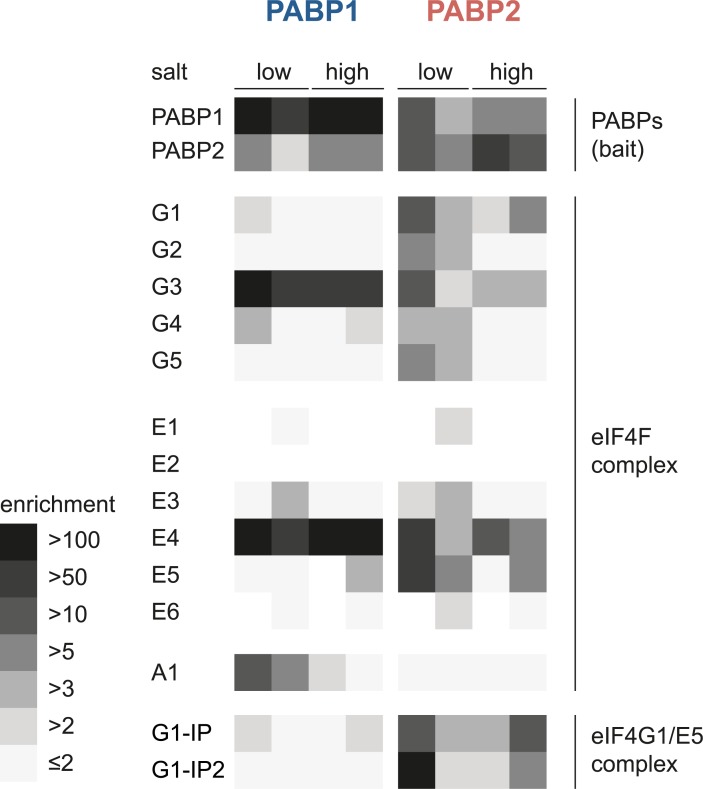
Interactions between PABPs and proteins involved in translation initiation. For each paralog of the eIF4F complex, the two PABPs and the two known interactors of the eIF4G1/eIF4E5 complex [41] the enrichment ratios from all four individual PABP pull-down experiments (low and high salt buffer) are illustrated by a heat map. No square is shown if the protein was not detected in the respective PABP pull-down replicate.

The data above confirm the strong association of PABP1 with eIF4G3 and eIF4E4. PABP2 in contrast interacts with eIF4E4, eIF4G3, eIF4G1 and two further proteins of the eIF4G1/eIF4E4 complex indicating multiple binding abilities to different paralogs of the eIF4F complex. For a more comprehensive picture, we analysed the enrichment ratios for all members of the translation initiation complex of the four individual experiments (Fig 4). PABP1 shows strong interactions with eIF4G3, eIF4E4 in all four experiments and, in particular in low salt conditions, also interaction with eIF4A1. Interactions with other translation initiation factors and with the two proteins known to interact with eIF4G1 and eIF4E5 [41] are absent or have very small enrichment factors. In contrast, PABP2 co-precipitated all five isoforms of eIF4G under low salt conditions and eIF4G1 and eIF4G3 also under high salt conditions. Similarly, all eIF4E subunits co-precipitated with PABP2 at least under low salt conditions, with the exception of eIF4E2 that has very low abundance [33]. Interestingly, both PABPs clearly co-precipitate each other, indicating that there may be complexes containing both PABPs on the same mRNA protein complex. For *Leishmania* PABPs such an interdependency has not been observed [36].

### Conclusions

Our data contribute towards better understanding of translation initiation control mechanisms in trypanosomes. Demonstration of highly distinct interactomes for the two paralogs of PABP in African trypanosomes indicates discrete functions.

Specifically, PABP1 has a small interactome, comprising eIF4E4 and eIF4G3, and the hypothetical RNA-binding proteins RBP23 and Tb927.7.7460. PABP1, eIF4E4, eIF4G3 and RBP23 are largely absent from stress granules; the localisation of Tb927.7.7460 remains unknown. In contrast, PABP2 has a rather more extensive interactome that includes all proteins precipitated with PABP1, except RBP23, and most subunits of the eIF4F complex. PABP2 and the majority of its interaction partners localise to starvation stress granules.

**Fig 5:**
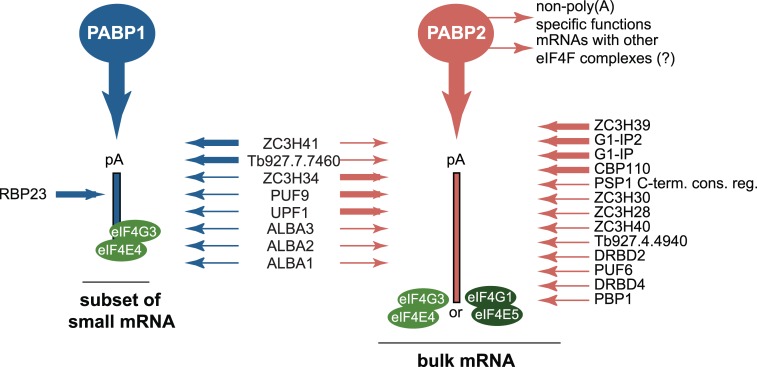
mRNA targets and interacting proteins of PABP1 and PABP2. Presented is a model that is consistent with all available data. PABP1 preferentially binds to a subset of mRNAs of mainly small size, while PABP2 binds to the larger set of bulk mRNAs. The basal translation initiation factors eIF4E4 and eIF4G3 associate with all mRNA targets, while eIF4E5 and eIF4G1 are specific for (a subset of?) PABP2 target mRNAs. All PABP interacting partners are shown with thick lines (>10 fold enrichment) or thin lines (>2 fold enrichment).

The impairment in stress granule localisation of the entire PABP1 complex challenges the previous assumption that this complex is the major translation initiation complex involved in bulk mRNA translation. At starvation, polysomes largely dissociate and most mRNAs and proteins involved in mRNA metabolism localise to starvation stress granules [44]. Localisation to granules is the default pathway, and impairment in stress granule localisation is the exception. As the PABP1/eIF4E4/eIF4G3 complex does not locate to stress granules, it is unlikely to regulate translation of bulk mRNAs. Instead, we propose that the PABP1/eIF4E4/eIF4G3 complex is specialised for the regulation of a small subgroup of mRNAs. It is tempting to speculate that this group of mRNAs could be those encoding ribosomal proteins, because these mRNAs are the only group of mRNAs that were found to be excluded from starvation stress granules [44] and these are of small size, consistent with a localisation of PABP1 to lower molecular weight polysomes [49]. The interaction of PABP2 with most eIF4F subunits and many mRNA metabolism proteins indicates a wider substrate specificity for this PABP subunit. The data are consistent with PABP2 being distributed over a range of different translation initiation complexes and mRNAs and thus being responsible for bulk mRNA translation. The eIF4F complex that was identified with highest confidence to bind to PABP2 is eIF4G1/eIF4E5 with its previously identified interactors G1-IP and G1-IP-2. The fact that both PABPs co-precipitate each other indicates that a separation of the two PABPs to a distinct group of mRNA targets is potentially not strict. A model of the PABP target mRNAs, consistent with the data, is shown in Fig 5. One limitation of this study is that only one life cycle stage, the procyclic stage, was examined and we can not exclude that the PABP interactomes and their localisations are different in other life cycle stages, for example in blood stream forms.

Notably, the functions of many eIF4F complex subunits (for example eIF4E1, 2, 6), and their association with the PABPs remains unsolved. The reason could be that all studies to date, including this one, focus only on the proliferating life cycle stages of the parasites. Translational control may, however, be particular important in G1-arrested stages or during differentiation processes and an analysis of these stages, *albeit* experimentally challenging, may be highly informative for a more comprehensive picture of the eIF4F/PABP complexes.

## Materials and methods

### Trypanosome work

*T*. *brucei* procyclic Lister 427 cells were cultured in SDM79 medium (containing fetal bovine serum from Sigma). The generation of transgenic trypanosomes was done using standard methods [65]. For starvation, parasites were washed once in one volume PBS and stored in PBS for two hours; the starvation time started at the first contact with PBS.

### Expression of fluorescently tagged proteins

Cell lines expressing PABP1-eYFP or PABP2-eYFP from endogenous loci were previously described [49]. Proteins were expressed as C-terminal (RBP23) or N-terminal (all others) eYFP fusion proteins by transfecting trypanosomes with PCR products obtained with the template plasmid pPOTv7-blast-blast-eYFP (RBP23) with oligonucleotides designed as described [66]. All transfected cell-lines co-expressed PABP2-mChFP from the endogenous locus [49] as a marker for starvation stress granules. The plasmid for the expression of a C-terminal 4Ty1 fusion protein was previously described for PABP1 [49]) and made accordingly for PABP2 [67].

### Fluorescence microscopy

Cells were washed with serum-free SDM79, fixed with 2.4% paraformaldehyde overnight, washed once in PBS and stained with 4′,6-diamidino-2-phenylindole (DAPI). Z-stacks (100 images, 100-nm spacing) were recorded with a custom-built TILL Photonics iMIC microscope equipped with a 100×, 1.4 numerical aperture objective (Olympus, Tokyo, Japan) and a sensicam qe CCD camera (PCO, Kehlheim, Germany) using exposure times of 500 ms for fluorescent proteins and 50 ms for DAPI. Images were deconvolved using Huygens Essential software (SVI, Hilversum, The Netherlands) and are presented as Z-projections (method sum slices) produced by ImageJ [68].

### Affinity isolation of PABP complexes

Procyclic trypanosomes were grown to a density of 5–8 x 10^6^ cells/ml. Four litre cultures were harvested in a F14S-6x 250 Y rotor at 1500g at room temperature in four subsequent centrifugations and washed once with 250 ml serum free SDM-79. Finally, the cells were sedimented by centrifugation (1500*g) into a capped 20 ml syringe placed in a 50 ml Falcon tube. After discarding all supernatant, inserting the plunger and removing the cap the cells were passed slowly into liquid nitrogen in order to form small pellets suitable for subsequent cryomilling. Frozen cells were processed by cryomilling into a fine powder in a planetary ball mill (Retsch) [55]. For precipitation, aliquots of approximately 50 mg powder (corresponding to ~2 x 10^8^ cells) were mixed with 1 ml ice-cold buffer (low salt buffer: 20 mM HEPES pH 7.4, 50 mM NaCl, 1 mM MgCl_2_, 100 μM CaCl_2_, 0.1% CHAPS; high salt buffer: 20 mM HEPES pH 7.4, 50 mM NaCl, 1 mM MgCl_2_, 100 μM CaCl_2_, 150 mM KCl, 0.1% CHAPS) complemented with protease inhibitors (Complete Protease Inhibitor Cocktail Tablet, EDTA-free, Roche). After sonication with a microtip sonicator (Misonix Utrasonic Processor XL) at setting 4 (~20 W output) for 2 x 1 second, insoluble material was removed by centrifugation (20,000 g, 10 min, 4°C). The clear lysate was incubated with 3 μl polyclonal anti-GFP llama antibodies covalently coupled to surface-activated Epoxy magnetic beads (Dynabeads M270 Epoxy, ThermoFisher) for two hours on a rotator. Beads were washed three times in the respective buffer (low salt or high salt buffer) and finally incubated in 15 μl 4 x NuPAGE LDS sample buffer (ThermoFisher), supplemented with 2 mM dithiothreitol, at 72°C for 15 minutes to elute the proteins. The precipitates were analysed on an SDS-PAGE gel stained with Coomassie. For subsequent proteomics analysis six pullout samples were pooled after the final washing step and eluted in 30 μl 4 x NuPAGE LDS Sample buffer, then run 1.5 cm into a NuPAGE Bis-Tris 4–12% gradient polyacrylamide gel (ThermoFisher) under reducing conditions. The respective gel region was sliced out and subjected to tryptic digest and reductive alkylation.

### Affinity isolation of eIF4E and G1-IP2 complexes

For the precipitation of eIF4E4 and G1-IP2, essentially the same protocol was used starting from 2 L cultures at a density 8 x 10^6^ cells/ml. The immunoprecipitation was carried out in low salt buffer using 5 ul recombinant, monoclonal dimeric fusion anti-GFP nanobody LaG16-LaG2 [69] coupled to magnetic beads. The same beads, where the antibody coupling step was omitted were used as a control. Eluates were run on a NuPAGE Bis-Tris 4–12% gradient polyacrylamide gel (ThermoFisher) under reducing conditions, then subjected to western blotting using standard procedures. 4Ty1 tagged fusion proteins were decorated with monoclonal anti-Ty1 antibody clone BB2 (Sigma) at 1:10,000 dilution. Quantitation was performed on raw images gathered under nonsaturating conditions using ImageJ [68] and enrichment ratios calculated comparing against uncoupled control beads.

### Mass spectrometry

Liquid chromatography tandem mass spectrometry (LC-MS^2^) was performed on a Dionex UltiMate 3000 RSLCnano System (Thermo Scientific, Waltham, MA, USA) coupled to an Orbitrap VelosPro mass spectrometer (Thermo Scientific) at the University of Dundee FingerPrints Proteomics facility and mass spectra analysed using MaxQuant version 1.5 [56] searching the *T*. *brucei brucei* 927 annotated protein database (release 8.1) from TriTrypDB [70]. Minimum peptide length was set at six amino acids, isoleucine and leucine were considered indistinguishable and false discovery rates (FDR) of 0.01 were calculated at the levels of peptides, proteins and modification sites based on the number of hits against the reversed sequence database. Ratios were calculated from label-free quantification intensities using only peptides that could be uniquely mapped to a given protein. If the identified peptide sequence set of one protein contained the peptide set of another protein, these two proteins were assigned to the same protein group. The mass spectrometry proteomics data have been deposited to the ProteomeXchange Consortium via the PRIDE [71]ƒ partner repository with the dataset identifier PXD008839.

## Supporting information

S1 Table Mass spectrometry data. **(A)** Raw data: all proteins identified in at least one of the experiment are shown with all parameters.
**(B)** List of proteins that were identified by more than two unique peptides in at least one of the experiments
**(C)** List of proteins that are at least two-fold enriched in both replicates of the PABP1 pull-down done in low salt buffer
**(D)** List of proteins that are at least two-fold enriched in both replicates of the PABP1 pull-down done in high salt buffer
**(E)** List of proteins that are at least two-fold enriched in both replicates of the PABP2 pull-down done in low salt buffer
**(F)** List of proteins that are at least two-fold enriched in both replicates of the PABP2 pull-down done in high salt buffer
**(G)** High confidence list of proteins significantly enriched in either PABP1 or PABP2 pull-downs or in both. The table contains the average enrichment ratios for the individual PABPs and the comparison between PABP1 and PABP2 (PABP1/PABP2). All ratios ≥2 are shown in bold. Localisation to starvation stress granules indicated with respective reference.
(XLSX)

S1 Fig Broad-field images of the cells shown in Fig 3. Broad-field images of untreated and starved (120 min PBS) trypanosomes expressing PABP2-mChFP as a stress granule marker together with the eYFP fusions of ZC3H41 (A), ZC3H40 (B), Tb927.11.14750 (C), RBP23 (D) or CBP110 (E). All images are presented as Z-stack projections (method sum slices) and at least two clonal cell lines gave identical localisations. Note that for RBP23 we observed differences in expression levels between cells that appeared not to correlate to the cell cycle; this was the case in all three clonal cell lines analysed.
(PDF)
