# Supplementary material for: Comparative proteomics of the two T. brucei PABPs suggests that PABP2 controls bulk mRNA
Source: PLoS Negl Trop Dis. 2018 Jul 24;12(7):e0006679. doi: 10.1371/journal.pntd.0006679 (PMC6075789; doi:10.1371/journal.pntd.0006679)
Supplement: S1 Fig — Broad-field images of untreated and starved (120 min PBS) trypanosomes expressing PABP2-mChFP as a stress granule marker together with the eYFP fusions of ZC3H41 (A), ZC3H40 (B), Tb927.11.14750 (C), RBP23 (D) or CBP110 (E). All images are presented as Z-stack projections (method sum slices) and at least two clonal cell lines gave identical localisations. Note that for RBP23 we observed differences in expression levels between cells that appeared not to correlate to the cell cycle; this was the case in all three clonal cell lines analysed. (PDF) [file pntd.0006679.s002.pdf]

S1 Figure A

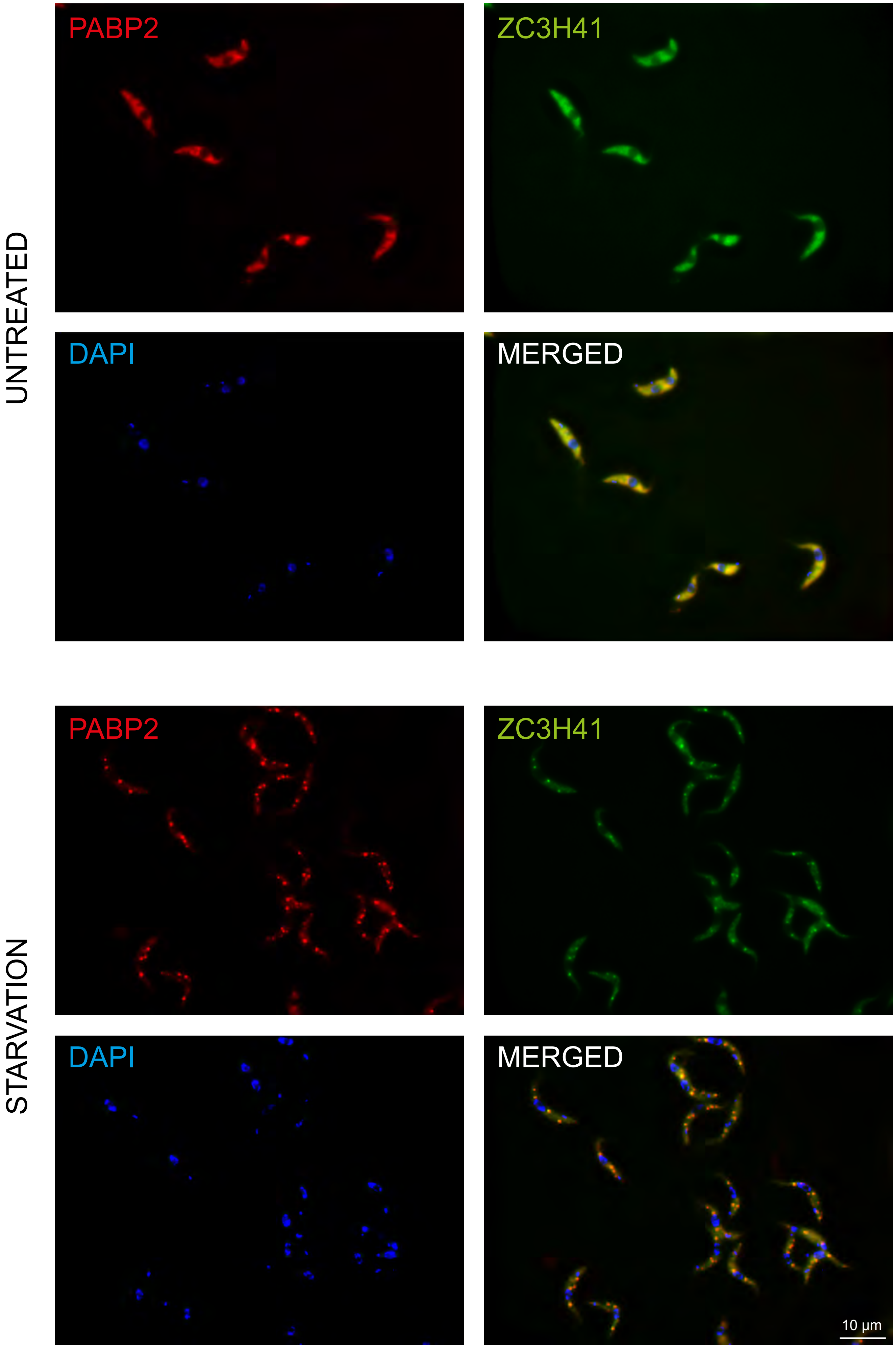
UNTREATED
PABP2
ZC3H41
DAPI
MERGED
STARVATION
PABP2
ZC3H41
DAPI
MERGED
10 µm

S1 Figure B

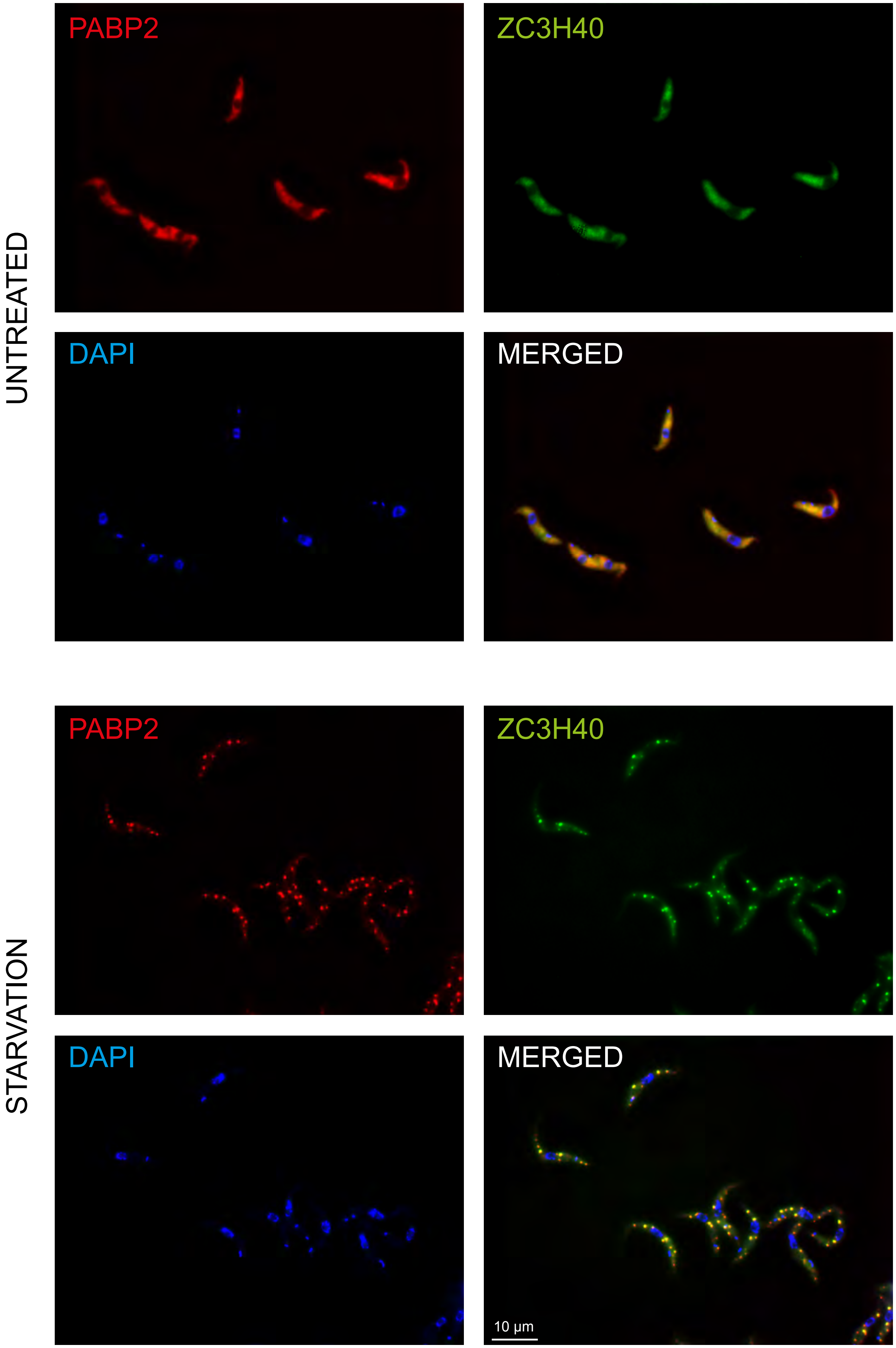
UNTREATED
PABP2
ZC3H40
DAPI
MERGED
STARVATION
PABP2
ZC3H40
DAPI
MERGED
10 µm

S1 Figure C

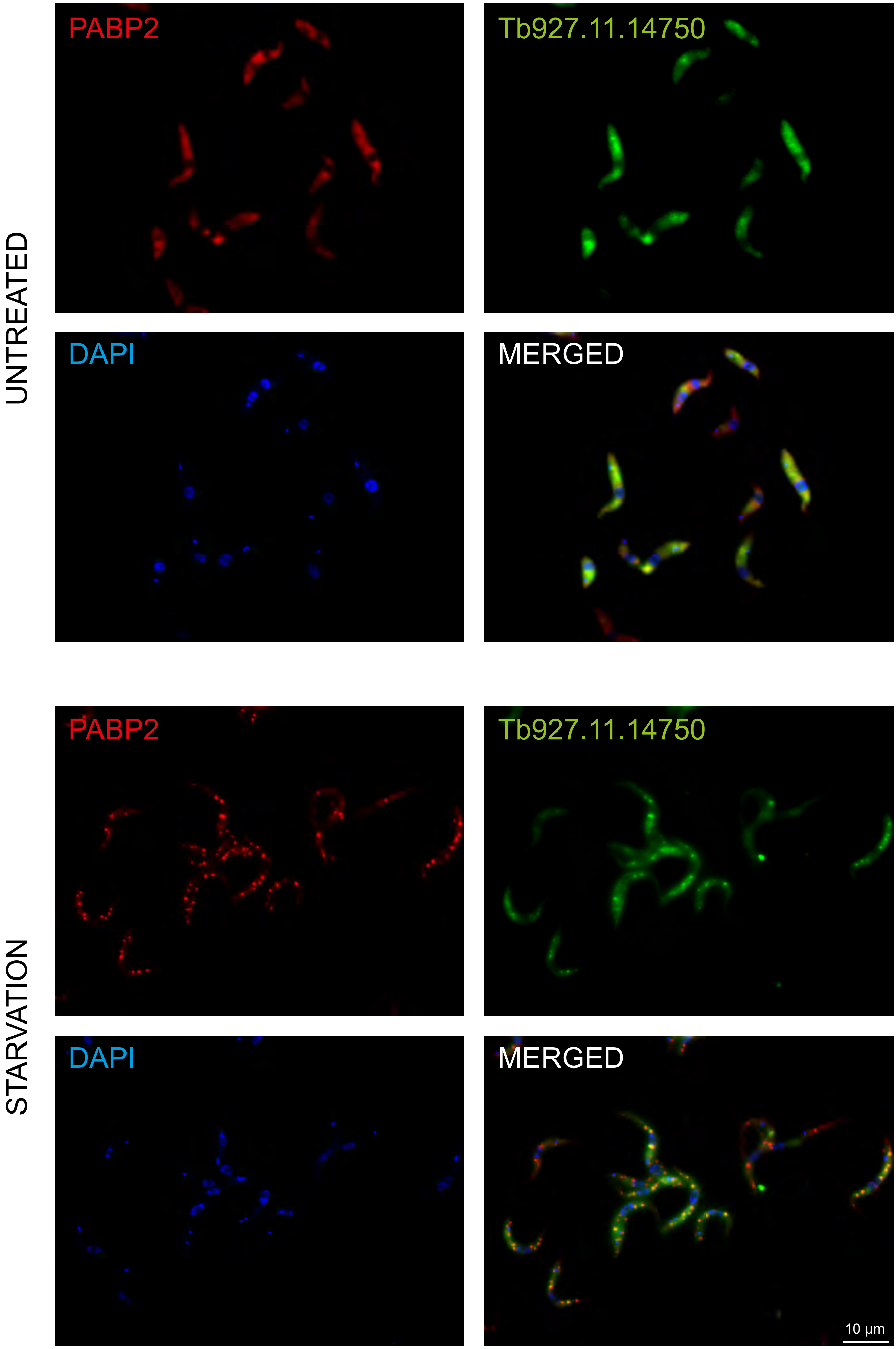
PABP2
Tb927.11.14750
DAPI
MERGED
UNTREATED
PABP2
Tb927.11.14750
DAPI
MERGED
STARVATION
10 µm

S1 Figure D

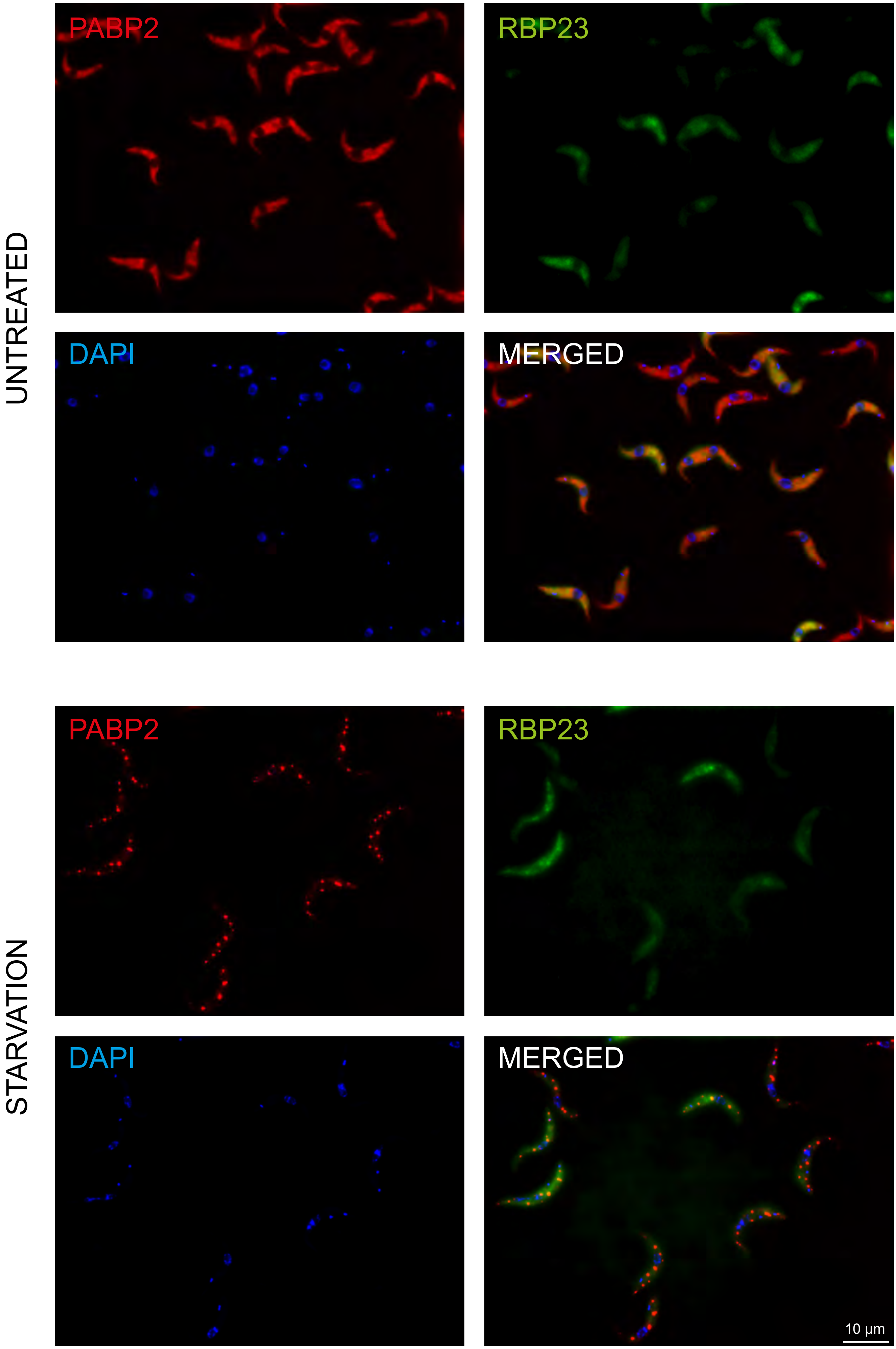
UNTREATED
PABP2
RBP23
DAPI
MERGED
STARVATION
PABP2
RBP23
DAPI
MERGED
10 µm

S1 Figure E

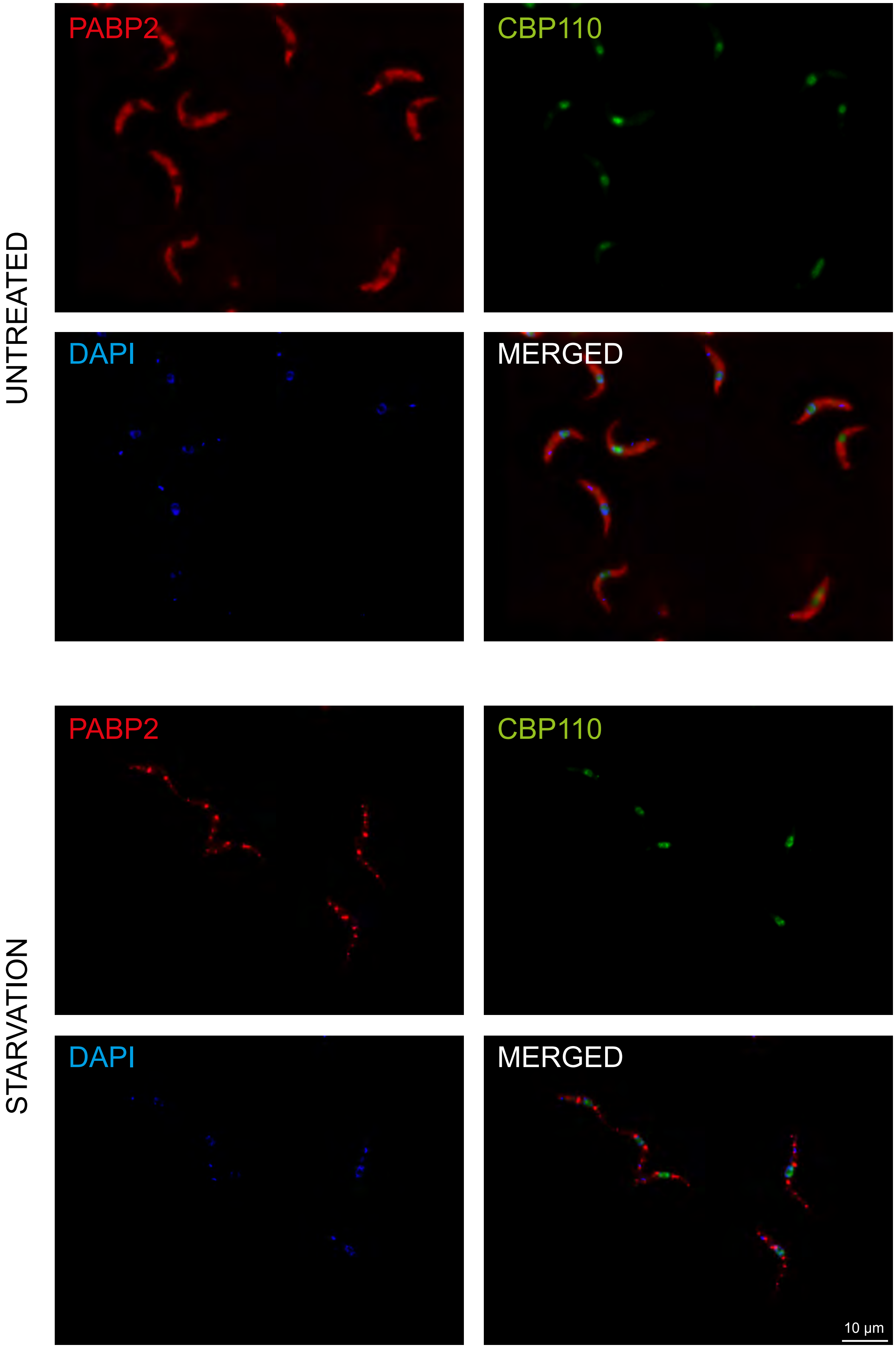
UNTREATED
PABP2
CBP110
DAPI
MERGED
STARVATION
PABP2
CBP110
DAPI
MERGED
10 µm

S1 Figure

Broad field images of untreated and starved (120 min PBS) trypanosomes expresing PABP2-mChFP as a stress granule marker together with the eYFP fusions of ZC3H41 (A), ZC3H40 (B), Tb927.11.14750 (C), RBP23 (D) orCBP110 (E). All images are presented as Z-stack projections (method sum slices) and at least 2 clonal cell lines gave identical localisations. Note that for RBP23 we observed differences in expression levels between cells that appeared not to correlate to the cell cycle; this was the case in all three clonal cell lines that were analysed.
